# Tetra­aqua­bis­(thio­urea-κ*S*)cadmium(II) triaqua­tris(thio­urea-κ*S*)cadmium(II) disulfate

**DOI:** 10.1107/S1600536812026682

**Published:** 2012-06-20

**Authors:** Masood Parvez, Farideh Jalilehvand, Zahra Amini

**Affiliations:** a Department of Chemistry, The University of Calgary, 2500 University Drive NW, Calgary, Alberta, Canada T2N 1N4

## Abstract

The title compound, [Cd(CH_4_N_2_S)_2_(H_2_O)_4_][Cd(CH_4_N_2_S)_3_(H_2_O)_3_](SO_4_)_2_, contains two mol­ecules of each of the Cd complexes and four sulfate ions in the asymmetric unit: all the Cd atoms exhibit distorted octa­hedral geometries. The Cd—S and Cd—O bond lengths around the Cd atoms in the bis­(thio­urea) cations are in the ranges 2.580 (4)–2.599 (4) and 2.323 (8)–2.421 (9) Å, respectively, and the S atoms are in a *cis* orientation. In the tris­(thio­urea) cations, the corresponding bond lengths around the Cd atoms are slightly longer and are in the ranges 2.559 (4)–2.706 (3) and 2.303 (7)–2.480 (10) Å, respectively, and the S atoms are in a *fac* disposition. The crystal structure features numerous N—H⋯O, N—H⋯N, O—H⋯O and O—H⋯N hydrogen bonds. Two O atoms of a sulfate anion were found to be disordered over two orientations in a 0.620 (9):0.380 (9) ratio. The crystal studied was a racemic twin with BASF = 0.17 (5)

## Related literature

For the structures of other cadmium–sulfate–thio­urea compounds, see: Cavaica *et al.* (1970 ▶); Corao & Baggio (1969 ▶); Oussaid *et al.* (2000 ▶). For the NMR measurement, see: Jalilehvand *et al.* (2012 ▶).
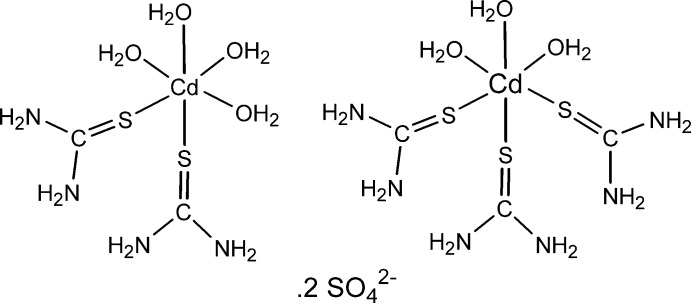


## Experimental

### 

#### Crystal data

[Cd(CH_4_N_2_S)_2_(H_2_O)_4_][Cd(CH_4_N_2_S)_3_(H_2_O)_3_](SO_4_)_2_
*M* *_r_* = 923.64
Monoclinic, 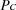

*a* = 10.9941 (3) Å
*b* = 11.7602 (3) Å
*c* = 24.0100 (5) Å
β = 98.9169 (12)°
*V* = 3066.80 (13) Å^3^
*Z* = 4
Mo *K*α radiation
μ = 1.94 mm^−1^
*T* = 173 K
0.07 × 0.06 × 0.05 mm

#### Data collection

Nonius KappaCCD diffractometer
Absorption correction: multi-scan (*SORTAV*; Blessing, 1997 ▶) *T*_min_ = 0.876, *T*_max_ = 0.909
16186 measured reflections
9995 independent reflections
8817 reflections with *I* > 2σ(*I*)
*R*_int_ = 0.037

#### Refinement

*R*[*F* ^2^ > 2σ(*F* ^2^)] = 0.047
*wR*(*F* ^2^) = 0.099
*S* = 1.08
9995 reflections
453 parameters
2 restraints
H-atom parameters constrained
Δρ_max_ = 0.78 e Å^−3^
Δρ_min_ = −0.64 e Å^−3^

### 

Data collection: *COLLECT* (Hooft, 1998 ▶); cell refinement: *DENZO* (Otwinowski & Minor, 1997 ▶); data reduction: *SCALEPACK* (Otwinowski & Minor, 1997 ▶); program(s) used to solve structure: *SHELXS97* (Sheldrick, 2008 ▶); program(s) used to refine structure: *SHELXL97* (Sheldrick, 2008 ▶); molecular graphics: *ORTEP-3 for Windows* (Farrugia, 1997 ▶); software used to prepare material for publication: *SHELXL97*.

## Supplementary Material

Crystal structure: contains datablock(s) global, I. DOI: 10.1107/S1600536812026682/hb6807sup1.cif

Structure factors: contains datablock(s) I. DOI: 10.1107/S1600536812026682/hb6807Isup2.hkl

Additional supplementary materials:  crystallographic information; 3D view; checkCIF report

## Figures and Tables

**Table 1:** Hydrogen-bond geometry (Å, °)

*D*—H⋯*A*	*D*—H	H⋯*A*	*D*⋯*A*	*D*—H⋯*A*
N1—H1*A*⋯O26^i^	0.88	2.14	2.958 (16)	154
N1—H1*A*⋯O26′^i^	0.88	2.24	3.11 (2)	168
N1—H1*B*⋯O17^i^	0.88	2.10	2.927 (15)	156
N1—H1*B*⋯O15^i^	0.88	2.55	3.111 (14)	122
N2—H2*A*⋯O24^i^	0.88	2.08	2.934 (15)	163
N2—H2*B*⋯O1	0.88	2.02	2.875 (14)	165
N3—H3*A*⋯O27	0.88	2.14	3.014 (14)	169
N4—H4*A*⋯O28	0.88	2.17	3.000 (13)	157
N4—H4*B*⋯O3	0.88	2.21	3.028 (12)	155
N4—H4*B*⋯O2	0.88	2.63	3.183 (12)	121
O1—H11⋯O29^ii^	0.82	1.96	2.743 (13)	160
O1—H12⋯O14	0.82	1.97	2.770 (12)	164
O2—H21⋯O26^ii^	0.82	2.05	2.636 (12)	128
O2—H21⋯O26′^ii^	0.82	2.12	2.856 (18)	150
O3—H32⋯O18^ii^	0.82	1.91	2.679 (10)	156
O4—H41⋯O15^ii^	0.82	1.88	2.691 (13)	172
O4—H42⋯O29^ii^	0.81	2.16	2.973 (12)	173
N5—H5*A*⋯O24^i^	0.88	2.13	3.000 (11)	171
N6—H6*A*⋯O23^i^	0.88	2.14	2.986 (11)	162
N6—H6*B*⋯O7	0.88	2.17	2.982 (12)	153
N6—H6*B*⋯O6	0.88	2.59	3.157 (12)	123
N7—H7*A*⋯O27	0.88	2.08	2.938 (17)	165
N7—H7*B*⋯O5	0.88	2.06	2.915 (14)	163
N8—H8*A*⋯O30	0.88	2.14	2.989 (15)	162
N8—H8*B*⋯O21^iii^	0.88	2.10	2.932 (15)	159
N8—H8*B*⋯O19^iii^	0.88	2.65	3.223 (14)	124
O5—H52⋯O11	0.82	2.09	2.887 (13)	164
O5—H51⋯N7	0.85	2.46	2.915 (14)	114
O6—H62⋯O30^iv^	0.83	1.84	2.641 (11)	162
O7—H72⋯O22^v^	0.82	1.95	2.718 (11)	155
O8—H81⋯O16^iv^	0.83	1.96	2.756 (12)	162
O8—H82⋯O10^iii^	0.81	2.18	2.922 (13)	152
N9—H9*A*⋯O21	0.88	2.40	3.150 (17)	143
N9—H9*A*⋯O20	0.88	2.50	3.320 (16)	156
N9—H9*B*⋯O27	0.88	1.98	2.834 (17)	164
N10—H10*A*⋯O21	0.88	2.26	3.040 (17)	148
N10—H10*B*⋯O10	0.88	2.10	2.954 (15)	163
N11—H11*A*⋯O18^i^	0.88	1.98	2.855 (12)	177
N11—H11*B*⋯O9	0.88	2.13	2.994 (12)	167
N12—H12*A*⋯O17^i^	0.88	1.93	2.797 (12)	169
N13—H13*A*⋯O2^iv^	0.88	2.36	3.193 (11)	158
N13—H13*B*⋯O28^iv^	0.88	1.98	2.848 (13)	168
N14—H14*A*⋯O26^i^	0.88	2.22	2.980 (13)	145
O9—H91⋯O28^iv^	0.82	2.03	2.810 (12)	159
O9—H92⋯O20^iv^	0.82	1.94	2.721 (11)	159
O9—H92⋯O22^iv^	0.82	2.54	3.189 (10)	137
O10—H101⋯O9	0.81	2.67	3.331 (11)	139
O10—H102⋯O25′^iv^	0.82	1.77	2.57 (2)	164
O10—H102⋯O25^iv^	0.82	1.99	2.761 (13)	156
O11—H111⋯O25^iv^	0.82	2.15	2.851 (14)	143
O11—H111⋯O26′^iv^	0.82	2.18	2.972 (18)	163
O11—H112⋯O29^iv^	0.82	1.96	2.718 (13)	153
N15—H15*A*⋯O30	0.88	2.44	3.222 (12)	148
N15—H15*A*⋯N8	0.88	2.69	3.331 (15)	131
N16—H16*A*⋯O6^vi^	0.88	2.34	3.184 (11)	161
N16—H16*B*⋯O23^ii^	0.88	1.99	2.866 (11)	172
N17—H17*A*⋯O21^iii^	0.88	1.99	2.864 (13)	176
N18—H18*A*⋯O22^iii^	0.88	1.98	2.846 (12)	169
N18—H18*B*⋯O12	0.88	2.15	2.998 (11)	163
N19—H19*A*⋯O17^vii^	0.88	2.42	3.174 (15)	145
N19—H19*A*⋯O16^vii^	0.88	2.43	3.258 (15)	156
N19—H19*B*⋯O24^i^	0.88	2.03	2.887 (15)	163
N19—H19*B*⋯O25′^i^	0.88	2.65	3.27 (2)	129
N20—H20*A*⋯O17^vii^	0.88	2.21	3.018 (16)	153
N20—H20*B*⋯O13	0.88	2.11	2.966 (15)	165
O12—H121⋯O23^ii^	0.82	2.04	2.846 (10)	167
O12—H122⋯O16^viii^	0.81	2.06	2.740 (9)	140
O13—H131⋯O19^ii^	0.82	1.88	2.694 (12)	171
O13—H132⋯O4^iii^	0.82	2.23	2.981 (12)	153
O14—H141⋯O20^ii^	0.82	1.90	2.714 (11)	173
O14—H142⋯O25^ii^	0.82	2.10	2.750 (14)	135
O14—H142⋯O25′^ii^	0.82	2.00	2.79 (2)	161

Symmetry codes: (i) 
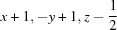
; (ii) 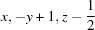; (iii) 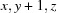; (iv) 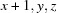; (v) 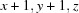; (vi) 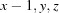; (vii) 
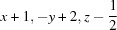
; (viii) 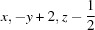.

**Fig. 1.**
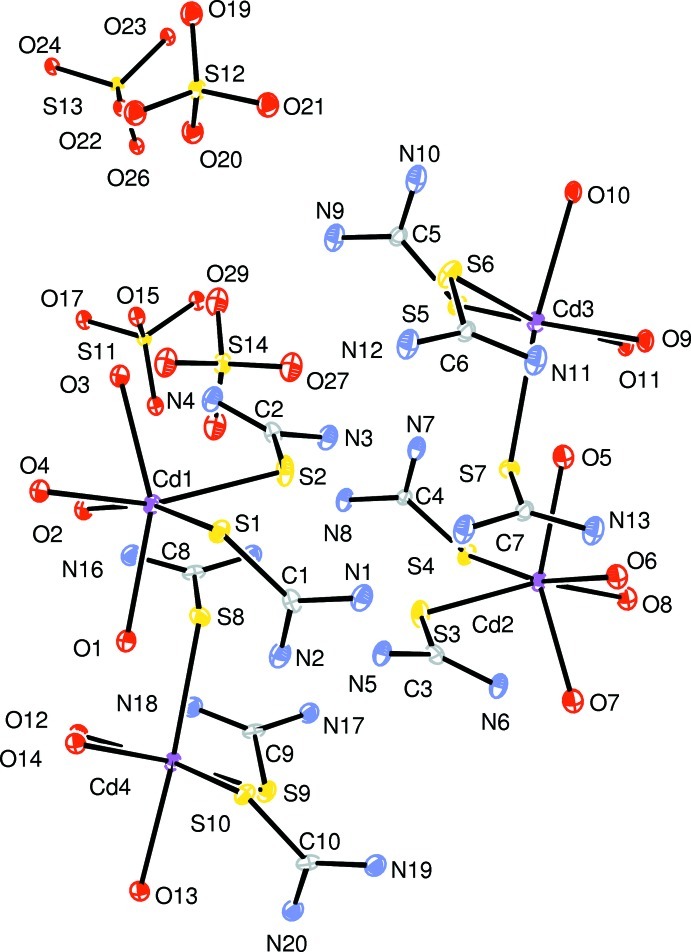
The molecular structure of the contents of an asymmetric unit of the title compound with displacement ellipsoids drawn at the 30% probability level.

**Fig. 2.**
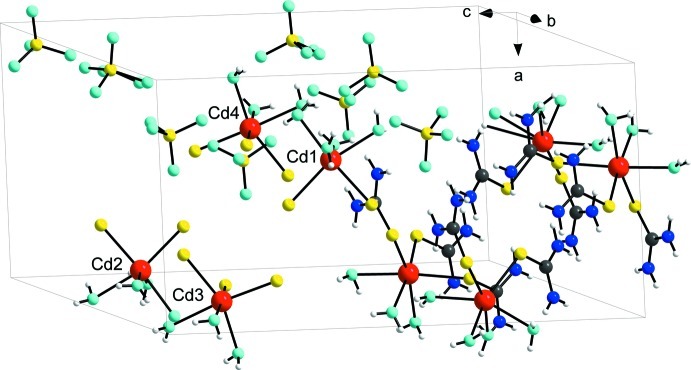
The content of a unit cell. For the cadmium complexes in the left part of the cell only the sulfur atoms are shown for clarity.

## supplementary crystallographic information

### Comment

A survey in the crystal structure database shows that mixing cadmium sulfate (CdSO_4_) with thiourea (TU) in the mole ratio 1:3 results in either monomeric [Cd(TU)_3_(SO_4_)] (Oussaid *et al.*, 2000; Cavaica *et al.*, 1970), (or dimeric [Cd(µ-TU)(TU)_2_(SO_4_)]_2_ complexes (Corao & Baggio, 1969) with the cadmium ion coordinating four (CdS_3_O) or five (CdS_4_O) ligand atoms, respectively.

With intention to prepare the mononuclear [Cd(TU)_3_(SO_4_)] complex for a solid state ^113^Cd NMR measurement (Jalilehvand *et al.*, 2012), a solution was prepared of a mixture of CdSO_4_^.^8/3H_2_O and thiourea in 1:3.8 mole ratio in hot water, and slowly evaporated resulting in colorless crystals. Elemental analysis of a ground sample *(i)* used for the solid state ^113^Cd NMR spectroscopy showed that the bulk of the crystalline solid mainly consisted of the [Cd(TU)_3_(SO_4_)] complex (see Special details section). However, elemental analyses of two random samples of the colorless crystals *(ii* and *iii)* showed that the sample was inhomogeneous. An X-ray crystallographic structure determination of the colorless crystal, revealed that *cis*-[Cd(TU)_2_(H_2_O)_4_]^2+^ and *fac*-[Cd(TU)_3_(H_2_O)_3_]^2+^ complexes had co-crystallized in the crystal. To our knowledge, this is the first report on the structure of hydrated Cd(II) thiourea complexes.

The asymmetric unit of the title co-crystal contains two cadmium(II) complexes of each type together with four sulfate ions (Fig. 1). All Cd atoms exhibit distorted octahedral geometry. The Cd–S and Cd–O distances around Cd1 and Cd2 atoms in the bis(thiourea) complex, *cis*-[Cd(TU)_2_(H_2_O)_4_]^2+^, lie in the ranges 2.580 (4) - 2.599 (4) Å and 2.323 (8) - 2.421 (9) Å, respectively. In the tris(thiourea) complex, *fac*-[Cd(TU)_3_(H_2_O)_3_]^2+^, the corresponding bond lengths around Cd3 and Cd4 atoms are slightly longer and lie in the ranges 2.559 (4) - 2.706 (3) Å and 2.303 (7) - 2.480 (10) Å, respectively. The crystal structure is stabilized by strong hydrogen bonds (Tab. 1).

### Experimental

A colorless solution containing a mixture of CdSO_4_^.^8/3(H_2_O) (1.505 g, 5.87 mmol) and thiourea (1.340 g, 22.33 mmol) in hot water (10 ml) was prepared. Slow evaporation of this solution resulted in an inhomogeneous mixture of colorless crystals, mainly consisting of [Cd(TU)_3_(SO_4_)] complex, as well as *cis*-[Cd(TU)_2_(H_2_O)_4_](SO_4_) and *fac*-[Cd(TU)_3_(H_2_O)_3_](SO_4_) complexes that were co-crystallized in the same unit cell.

### Refinement

All H atoms were positioned geometrically and refined using a riding model, and the *U*_iso_(H) were allowed at 1.2*U*_eq_(parent atom). Water H-atoms were contrained at distances O—H = 0.82 Å and EADP commands were used to model the disorder. An absolute structure using Flack method was not determined as the crystals were composed of racemic twins with BASF = 0.17 (5); Fridel pairs were merged. Two oxygen atoms of a sulfate anion were disordered over two sites each in a ratio 0.620 (9):0.380 (9). A refinement of the structure with half the current length of the cell axis-*b*, allowing half of the contents of the unit cell, resulted in a grossly disordered model which was therefore ruled out as the unit cell. Therefore, the model was refined in the current supercell presented in this paper.

### Crystal data

**Table:**

[Cd(CH_4_N_2_S)_2_(H_2_O)_4_][Cd(CH_4_N_2_S)_3_(H_2_O)_3_](SO_4_)_2_	*F*(000) = 1848
*M**_r_* = 923.64	*D*_x_ = 2.000 Mg m^−^^3^
Monoclinic, *P**c*	Mo *K*α radiation, λ = 0.71073 Å
Hall symbol: P -2yc	Cell parameters from 2175 reflections
*a* = 10.9941 (3) Å	θ = 3.4–30.0°
*b* = 11.7602 (3) Å	µ = 1.94 mm^−^^1^
*c* = 24.0100 (5) Å	*T* = 173 K
β = 98.9169 (12)°	Prism, colorless
*V* = 3066.80 (13) Å^3^	0.07 × 0.06 × 0.05 mm
*Z* = 4	

### Data collection

**Table:**

Nonius KappaCCD diffractometer	9995 independent reflections
Radiation source: fine-focus sealed tube	8817 reflections with *I* > 2σ(*I*)
Graphite monochromator	*R*_int_ = 0.037
ω and φ scans	θ_max_ = 25.0°, θ_min_ = 2.4°
Absorption correction: multi-scan (*SORTAV*; Blessing, 1997)	*h* = −12→13
*T*_min_ = 0.876, *T*_max_ = 0.909	*k* = −13→13
16186 measured reflections	*l* = −28→28

### Refinement

**Table:**

Refinement on *F*^2^	Secondary atom site location: difference Fourier map
Least-squares matrix: full	Hydrogen site location: inferred from neighbouring sites
*R*[*F*^2^ > 2σ(*F*^2^)] = 0.047	H-atom parameters constrained
*wR*(*F*^2^) = 0.099	*w* = 1/[σ^2^(*F*_o_^2^) + (0.0102*P*)^2^ + 30.5291*P*] where *P* = (*F*_o_^2^ + 2*F*_c_^2^)/3
*S* = 1.08	(Δ/σ)_max_ = 0.004
9995 reflections	Δρ_max_ = 0.78 e Å^−^^3^
453 parameters	Δρ_min_ = −0.64 e Å^−^^3^
2 restraints	Absolute structure: Flack, H. D. (1983). *Acta Cryst.* A39, 876–881
Primary atom site location: structure-invariant direct methods	Flack parameter: 0.15 (5)

### Special details

**Table:**

Experimental. Elemental analysis of a ground sample *(i)* used for the solid state ^113^Cd NMR spectroscopy: C = 8.44%, H = 2.62%, N = 19.27% (calculated for [Cd(TU)_3_(SO_4_)]: CdC_3_H_12_N_6_O_4_S_4_ (M.W. = 436.7), C = 8.24%, H = 2.75%, N = 19.23%). Elemental analyses of two random samples of the colorless crystals: *(ii)* exp.: C = 8.23%, H = 2.68%, N = 19.08%, and *(iii)* exp.: C = 7.44%, H = 3.01%, N = 17.22% (calculated for [Cd(TU)_3_(H_2_O)_3_](SO_4_): CdC_3_H_18_N_6_O_7_S_4_ (M.W. = 490.7), C = 7.33%, H = 3.67%, N = 17.12%).
Geometry. All e.s.d.'s (except the e.s.d. in the dihedral angle between two l.s. planes) are estimated using the full covariance matrix. The cell e.s.d.'s are taken into account individually in the estimation of e.s.d.'s in distances, angles and torsion angles; correlations between e.s.d.'s in cell parameters are only used when they are defined by crystal symmetry. An approximate (isotropic) treatment of cell e.s.d.'s is used for estimating e.s.d.'s involving l.s. planes.

### Fractional atomic coordinates and isotropic or equivalent isotropic displacement parameters (Å^2^)

**Table:**

	*x*	*y*	*z*	*U*_iso_*/*U*_eq_	Occ. (<1)
Cd1	0.43936 (7)	0.41388 (8)	0.00967 (3)	0.0175 (3)	
S1	0.6026 (4)	0.3423 (3)	−0.04845 (17)	0.0207 (9)	
S2	0.6146 (3)	0.4338 (3)	0.09512 (14)	0.0282 (6)	
C1	0.7138 (13)	0.4492 (12)	−0.0421 (5)	0.0176 (17)	
C2	0.5530 (9)	0.4611 (8)	0.1553 (4)	0.0176 (17)	
N1	0.8306 (12)	0.4197 (10)	−0.0393 (5)	0.0261 (12)	
H1A	0.8882	0.4724	−0.0362	0.031*	
H1B	0.8509	0.3475	−0.0405	0.031*	
N2	0.6847 (11)	0.5571 (11)	−0.0403 (5)	0.0261 (12)	
H2A	0.7428	0.6093	−0.0372	0.031*	
H2B	0.6071	0.5774	−0.0421	0.031*	
N3	0.6216 (8)	0.5129 (8)	0.1981 (4)	0.0261 (12)	
H3A	0.5922	0.5247	0.2297	0.031*	
H3B	0.6965	0.5356	0.1950	0.031*	
N4	0.4413 (8)	0.4275 (8)	0.1604 (4)	0.0261 (12)	
H4A	0.4124	0.4395	0.1921	0.031*	
H4B	0.3953	0.3930	0.1321	0.031*	
O1	0.4217 (8)	0.5795 (8)	−0.0511 (4)	0.0227 (9)	
H11	0.4015	0.5634	−0.0844	0.027*	
H12	0.3894	0.6411	−0.0460	0.027*	
O2	0.2817 (7)	0.5077 (6)	0.0450 (3)	0.0227 (9)	
H21	0.2345	0.5180	0.0155	0.027*	
H22	0.3234	0.5657	0.0511	0.027*	
O3	0.3575 (7)	0.2714 (6)	0.0616 (3)	0.0227 (9)	
H31	0.4142	0.2255	0.0699	0.027*	
H32	0.2932	0.2347	0.0591	0.027*	
O4	0.3053 (9)	0.3178 (7)	−0.0617 (4)	0.0227 (9)	
H41	0.2319	0.3144	−0.0588	0.027*	
H42	0.3123	0.3608	−0.0875	0.027*	
Cd2	0.94999 (7)	0.83626 (9)	0.28083 (3)	0.0174 (3)	
S3	0.7784 (3)	0.8140 (3)	0.19488 (14)	0.0285 (6)	
S4	0.7831 (4)	0.9044 (3)	0.33779 (17)	0.0196 (9)	
C3	0.8400 (10)	0.7856 (9)	0.1342 (4)	0.024 (2)	
C4	0.6721 (13)	0.7993 (12)	0.3289 (5)	0.015 (2)	
N5	0.7722 (8)	0.7352 (7)	0.0914 (4)	0.0238 (11)	
H5A	0.8036	0.7198	0.0607	0.029*	
H5B	0.6954	0.7168	0.0935	0.029*	
N6	0.9549 (8)	0.8122 (7)	0.1300 (4)	0.0238 (11)	
H6A	0.9846	0.7961	0.0989	0.029*	
H6B	1.0017	0.8459	0.1582	0.029*	
N7	0.7024 (10)	0.6918 (11)	0.3256 (5)	0.0238 (11)	
H7A	0.6452	0.6389	0.3227	0.029*	
H7B	0.7800	0.6726	0.3263	0.029*	
N8	0.5568 (12)	0.8291 (10)	0.3279 (5)	0.0238 (11)	
H8A	0.4989	0.7768	0.3250	0.029*	
H8B	0.5373	0.9013	0.3301	0.029*	
O5	0.9699 (8)	0.6725 (8)	0.3411 (4)	0.0275 (10)	
H51	0.9203	0.6571	0.3638	0.033*	
H52	1.0087	0.6135	0.3386	0.033*	
O6	1.1097 (7)	0.7376 (7)	0.2461 (3)	0.0275 (10)	
H61	1.0851	0.6714	0.2438	0.033*	
H62	1.1831	0.7350	0.2601	0.033*	
O7	1.0359 (7)	0.9713 (6)	0.2252 (3)	0.0275 (10)	
H71	1.0057	1.0222	0.2418	0.033*	
H72	1.1109	0.9747	0.2344	0.033*	
O8	1.0807 (10)	0.9404 (8)	0.3495 (4)	0.0275 (10)	
H81	1.0944	0.9406	0.3842	0.033*	
H82	1.0377	0.9954	0.3398	0.033*	
Cd3	0.95867 (7)	0.33264 (8)	0.27590 (3)	0.0184 (3)	
S5	0.7880 (4)	0.4025 (3)	0.32882 (17)	0.0185 (8)	
S6	0.8492 (3)	0.1824 (3)	0.20761 (14)	0.0264 (6)	
S7	0.9420 (3)	0.5165 (2)	0.20781 (14)	0.0225 (6)	
C5	0.6854 (14)	0.2918 (12)	0.3271 (6)	0.0196 (14)	
C6	0.9099 (10)	0.1920 (9)	0.1457 (4)	0.0196 (14)	
C7	0.9997 (10)	0.4949 (9)	0.1465 (4)	0.0196 (14)	
N9	0.5673 (12)	0.3151 (12)	0.3264 (5)	0.0306 (10)	
H9A	0.5142	0.2595	0.3277	0.037*	
H9B	0.5419	0.3861	0.3247	0.037*	
N10	0.7216 (11)	0.1847 (12)	0.3297 (5)	0.0306 (10)	
H10A	0.6675	0.1299	0.3309	0.037*	
H10B	0.7997	0.1680	0.3302	0.037*	
N11	1.0266 (9)	0.2179 (8)	0.1451 (4)	0.0306 (10)	
H11A	1.0559	0.2182	0.1130	0.037*	
H11B	1.0750	0.2349	0.1767	0.037*	
N12	0.8397 (9)	0.1671 (9)	0.0984 (4)	0.0306 (10)	
H12A	0.8702	0.1677	0.0666	0.037*	
H12B	0.7618	0.1497	0.0983	0.037*	
N13	1.1188 (9)	0.5005 (8)	0.1441 (4)	0.0306 (10)	
H13A	1.1453	0.4926	0.1116	0.037*	
H13B	1.1716	0.5121	0.1751	0.037*	
N14	0.9224 (9)	0.4772 (9)	0.0987 (4)	0.0306 (10)	
H14A	0.9512	0.4695	0.0667	0.037*	
H14B	0.8427	0.4731	0.0992	0.037*	
O9	1.1578 (7)	0.3016 (6)	0.2561 (3)	0.0205 (10)	
H91	1.1995	0.3594	0.2624	0.025*	
H92	1.1970	0.2495	0.2732	0.025*	
O10	0.9931 (7)	0.1753 (7)	0.3437 (4)	0.0205 (10)	
H101	1.0583	0.1809	0.3319	0.025*	
H102	1.0039	0.1975	0.3765	0.025*	
O11	1.0797 (9)	0.4490 (8)	0.3510 (4)	0.0205 (10)	
H111	1.0876	0.4327	0.3847	0.025*	
H112	1.1392	0.4880	0.3467	0.025*	
Cd4	0.42807 (7)	0.91384 (8)	0.01451 (3)	0.0159 (3)	
S8	0.4471 (3)	0.7294 (2)	0.08265 (13)	0.0222 (6)	
S9	0.5445 (3)	1.0590 (2)	0.08499 (14)	0.0226 (6)	
S10	0.6009 (4)	0.8462 (3)	−0.03811 (18)	0.0202 (9)	
C8	0.3906 (10)	0.7517 (8)	0.1463 (4)	0.0199 (14)	
C9	0.4804 (10)	1.0523 (8)	0.1473 (4)	0.0199 (14)	
C10	0.7041 (15)	0.9583 (12)	−0.0345 (6)	0.0199 (14)	
N15	0.4670 (9)	0.7666 (8)	0.1924 (4)	0.0249 (9)	
H15A	0.4394	0.7724	0.2248	0.030*	
H15B	0.5465	0.7708	0.1914	0.030*	
N16	0.2711 (9)	0.7451 (7)	0.1469 (4)	0.0249 (9)	
H16A	0.2422	0.7509	0.1790	0.030*	
H16B	0.2202	0.7350	0.1152	0.030*	
N17	0.5546 (9)	1.0710 (8)	0.1947 (4)	0.0249 (9)	
H17A	0.5261	1.0687	0.2269	0.030*	
H17B	0.6329	1.0857	0.1942	0.030*	
N18	0.3642 (8)	1.0303 (7)	0.1464 (4)	0.0249 (9)	
H18A	0.3334	1.0275	0.1781	0.030*	
H18B	0.3162	1.0181	0.1140	0.030*	
N19	0.8225 (12)	0.9344 (10)	−0.0319 (5)	0.0249 (9)	
H19A	0.8769	0.9898	−0.0296	0.030*	
H19B	0.8468	0.8632	−0.0324	0.030*	
N20	0.6692 (11)	1.0658 (10)	−0.0336 (5)	0.0249 (9)	
H20A	0.7245	1.1204	−0.0313	0.030*	
H20B	0.5908	1.0827	−0.0352	0.030*	
O12	0.2333 (7)	0.9439 (6)	0.0356 (3)	0.0181 (10)	
H121	0.1877	0.8906	0.0399	0.022*	
H122	0.2103	0.9712	0.0046	0.022*	
O13	0.3967 (7)	1.0792 (7)	−0.0483 (3)	0.0181 (10)	
H131	0.3797	1.0641	−0.0819	0.022*	
H132	0.3594	1.1377	−0.0428	0.022*	
O14	0.3135 (9)	0.7928 (7)	−0.0567 (4)	0.0181 (10)	
H141	0.3186	0.8041	−0.0898	0.022*	
H142	0.2419	0.8054	−0.0526	0.022*	
S11	0.0446 (4)	0.8135 (3)	0.48584 (15)	0.0170 (8)	
O15	0.0669 (7)	0.7164 (6)	0.4470 (3)	0.0171 (9)	
O16	0.0752 (6)	0.9236 (6)	0.4635 (3)	0.0171 (9)	
O17	−0.0875 (8)	0.8114 (7)	0.4923 (3)	0.0171 (9)	
O18	0.1206 (7)	0.7914 (6)	0.5408 (3)	0.0171 (9)	
S12	0.3433 (4)	0.0625 (3)	0.30619 (15)	0.0167 (8)	
O19	0.3147 (8)	−0.0269 (7)	0.3431 (4)	0.0285 (11)	
O20	0.3087 (8)	0.1721 (7)	0.3313 (3)	0.0285 (11)	
O21	0.4753 (9)	0.0635 (8)	0.3028 (4)	0.0285 (11)	
O22	0.2695 (8)	0.0525 (7)	0.2497 (4)	0.0285 (11)	
S13	0.0318 (3)	0.3167 (3)	0.49290 (15)	0.0148 (8)	
O23	0.1037 (7)	0.2637 (6)	0.5428 (3)	0.0135 (9)	
O24	−0.0988 (8)	0.2973 (7)	0.4912 (3)	0.0135 (9)	
O25	0.0799 (9)	0.2921 (9)	0.4416 (4)	0.0135 (9)	0.620 (9)
O26	0.0505 (9)	0.4439 (9)	0.5051 (4)	0.0135 (9)	0.620 (9)
O25'	0.0618 (16)	0.2170 (16)	0.4489 (7)	0.0135 (9)	0.380 (9)
O26'	0.0622 (15)	0.4256 (14)	0.4727 (7)	0.0135 (9)	0.380 (9)
S14	0.3566 (4)	0.5588 (3)	0.29742 (17)	0.0209 (8)	
O27	0.4891 (10)	0.5403 (9)	0.2982 (5)	0.0376 (11)	
O28	0.2885 (8)	0.5043 (7)	0.2466 (4)	0.0376 (11)	
O29	0.3164 (8)	0.5118 (7)	0.3478 (4)	0.0376 (11)	
O30	0.3337 (8)	0.6840 (7)	0.2958 (4)	0.0376 (11)	

### Atomic displacement parameters (Å^2^)

**Table:**

	*U*^11^	*U*^22^	*U*^33^	*U*^12^	*U*^13^	*U*^23^
Cd1	0.0161 (6)	0.0184 (5)	0.0178 (5)	−0.0019 (4)	0.0017 (5)	0.0003 (4)
S1	0.0178 (19)	0.0174 (16)	0.027 (2)	−0.0038 (13)	0.0040 (15)	−0.0045 (13)
S2	0.0147 (13)	0.0521 (18)	0.0180 (13)	−0.0012 (13)	0.0031 (11)	−0.0086 (13)
C1	0.018 (4)	0.024 (4)	0.010 (3)	0.004 (3)	0.001 (3)	−0.006 (3)
C2	0.018 (4)	0.024 (4)	0.010 (3)	0.004 (3)	0.001 (3)	−0.006 (3)
N1	0.020 (3)	0.035 (3)	0.024 (3)	−0.005 (2)	0.006 (2)	−0.004 (2)
N2	0.020 (3)	0.035 (3)	0.024 (3)	−0.005 (2)	0.006 (2)	−0.004 (2)
N3	0.020 (3)	0.035 (3)	0.024 (3)	−0.005 (2)	0.006 (2)	−0.004 (2)
N4	0.020 (3)	0.035 (3)	0.024 (3)	−0.005 (2)	0.006 (2)	−0.004 (2)
O1	0.018 (2)	0.024 (2)	0.025 (2)	−0.0018 (17)	0.0021 (17)	0.0031 (17)
O2	0.018 (2)	0.024 (2)	0.025 (2)	−0.0018 (17)	0.0021 (17)	0.0031 (17)
O3	0.018 (2)	0.024 (2)	0.025 (2)	−0.0018 (17)	0.0021 (17)	0.0031 (17)
O4	0.018 (2)	0.024 (2)	0.025 (2)	−0.0018 (17)	0.0021 (17)	0.0031 (17)
Cd2	0.0125 (6)	0.0234 (5)	0.0170 (5)	−0.0011 (4)	0.0046 (4)	−0.0009 (4)
S3	0.0166 (14)	0.0519 (19)	0.0171 (13)	0.0006 (13)	0.0028 (11)	−0.0040 (13)
S4	0.017 (2)	0.0209 (16)	0.0234 (18)	−0.0031 (14)	0.0098 (15)	−0.0071 (13)
C3	0.023 (6)	0.027 (6)	0.018 (5)	0.000 (5)	−0.004 (4)	0.006 (4)
C4	0.014 (5)	0.023 (5)	0.007 (4)	−0.004 (4)	0.002 (4)	0.001 (4)
N5	0.014 (3)	0.031 (3)	0.028 (3)	0.000 (2)	0.008 (2)	−0.004 (2)
N6	0.014 (3)	0.031 (3)	0.028 (3)	0.000 (2)	0.008 (2)	−0.004 (2)
N7	0.014 (3)	0.031 (3)	0.028 (3)	0.000 (2)	0.008 (2)	−0.004 (2)
N8	0.014 (3)	0.031 (3)	0.028 (3)	0.000 (2)	0.008 (2)	−0.004 (2)
O5	0.024 (2)	0.032 (2)	0.026 (2)	0.0030 (18)	0.0040 (18)	0.0001 (18)
O6	0.024 (2)	0.032 (2)	0.026 (2)	0.0030 (18)	0.0040 (18)	0.0001 (18)
O7	0.024 (2)	0.032 (2)	0.026 (2)	0.0030 (18)	0.0040 (18)	0.0001 (18)
O8	0.024 (2)	0.032 (2)	0.026 (2)	0.0030 (18)	0.0040 (18)	0.0001 (18)
Cd3	0.0166 (6)	0.0212 (5)	0.0174 (6)	0.0002 (4)	0.0027 (4)	−0.0015 (4)
S5	0.0170 (19)	0.0155 (15)	0.0241 (18)	−0.0021 (13)	0.0064 (14)	−0.0035 (12)
S6	0.0256 (15)	0.0354 (16)	0.0195 (13)	−0.0119 (12)	0.0079 (11)	−0.0114 (11)
S7	0.0198 (14)	0.0270 (14)	0.0210 (13)	−0.0006 (11)	0.0044 (11)	0.0063 (11)
C5	0.017 (3)	0.025 (3)	0.017 (3)	−0.004 (3)	0.002 (3)	0.002 (3)
C6	0.017 (3)	0.025 (3)	0.017 (3)	−0.004 (3)	0.002 (3)	0.002 (3)
C7	0.017 (3)	0.025 (3)	0.017 (3)	−0.004 (3)	0.002 (3)	0.002 (3)
N9	0.023 (2)	0.048 (3)	0.022 (2)	−0.008 (2)	0.0042 (17)	−0.0047 (18)
N10	0.023 (2)	0.048 (3)	0.022 (2)	−0.008 (2)	0.0042 (17)	−0.0047 (18)
N11	0.023 (2)	0.048 (3)	0.022 (2)	−0.008 (2)	0.0042 (17)	−0.0047 (18)
N12	0.023 (2)	0.048 (3)	0.022 (2)	−0.008 (2)	0.0042 (17)	−0.0047 (18)
N13	0.023 (2)	0.048 (3)	0.022 (2)	−0.008 (2)	0.0042 (17)	−0.0047 (18)
N14	0.023 (2)	0.048 (3)	0.022 (2)	−0.008 (2)	0.0042 (17)	−0.0047 (18)
O9	0.015 (2)	0.023 (2)	0.021 (2)	−0.0054 (19)	−0.0016 (18)	−0.0028 (19)
O10	0.015 (2)	0.023 (2)	0.021 (2)	−0.0054 (19)	−0.0016 (18)	−0.0028 (19)
O11	0.015 (2)	0.023 (2)	0.021 (2)	−0.0054 (19)	−0.0016 (18)	−0.0028 (19)
Cd4	0.0141 (6)	0.0188 (5)	0.0154 (5)	−0.0011 (4)	0.0039 (4)	−0.0014 (4)
S8	0.0228 (14)	0.0256 (14)	0.0175 (13)	0.0002 (11)	0.0012 (11)	0.0043 (11)
S9	0.0198 (14)	0.0319 (15)	0.0175 (12)	−0.0061 (11)	0.0070 (10)	−0.0024 (11)
S10	0.018 (2)	0.0168 (16)	0.0280 (19)	−0.0023 (13)	0.0086 (15)	−0.0014 (13)
C8	0.027 (4)	0.013 (3)	0.022 (3)	−0.002 (3)	0.011 (3)	0.002 (3)
C9	0.027 (4)	0.013 (3)	0.022 (3)	−0.002 (3)	0.011 (3)	0.002 (3)
C10	0.027 (4)	0.013 (3)	0.022 (3)	−0.002 (3)	0.011 (3)	0.002 (3)
N15	0.024 (2)	0.030 (2)	0.022 (2)	−0.0051 (18)	0.0038 (17)	0.0013 (17)
N16	0.024 (2)	0.030 (2)	0.022 (2)	−0.0051 (18)	0.0038 (17)	0.0013 (17)
N17	0.024 (2)	0.030 (2)	0.022 (2)	−0.0051 (18)	0.0038 (17)	0.0013 (17)
N18	0.024 (2)	0.030 (2)	0.022 (2)	−0.0051 (18)	0.0038 (17)	0.0013 (17)
N19	0.024 (2)	0.030 (2)	0.022 (2)	−0.0051 (18)	0.0038 (17)	0.0013 (17)
N20	0.024 (2)	0.030 (2)	0.022 (2)	−0.0051 (18)	0.0038 (17)	0.0013 (17)
O12	0.019 (2)	0.025 (2)	0.011 (2)	0.003 (2)	0.0043 (18)	−0.0006 (18)
O13	0.019 (2)	0.025 (2)	0.011 (2)	0.003 (2)	0.0043 (18)	−0.0006 (18)
O14	0.019 (2)	0.025 (2)	0.011 (2)	0.003 (2)	0.0043 (18)	−0.0006 (18)
S11	0.0145 (18)	0.0192 (15)	0.0169 (16)	0.0014 (13)	0.0007 (13)	−0.0014 (12)
O15	0.016 (2)	0.024 (2)	0.0121 (17)	0.0030 (16)	0.0037 (15)	−0.0031 (15)
O16	0.016 (2)	0.024 (2)	0.0121 (17)	0.0030 (16)	0.0037 (15)	−0.0031 (15)
O17	0.016 (2)	0.024 (2)	0.0121 (17)	0.0030 (16)	0.0037 (15)	−0.0031 (15)
O18	0.016 (2)	0.024 (2)	0.0121 (17)	0.0030 (16)	0.0037 (15)	−0.0031 (15)
S12	0.0132 (17)	0.0206 (15)	0.0173 (17)	0.0013 (13)	0.0056 (13)	−0.0020 (13)
O19	0.023 (2)	0.032 (2)	0.030 (2)	0.0016 (18)	0.0043 (18)	0.0011 (19)
O20	0.023 (2)	0.032 (2)	0.030 (2)	0.0016 (18)	0.0043 (18)	0.0011 (19)
O21	0.023 (2)	0.032 (2)	0.030 (2)	0.0016 (18)	0.0043 (18)	0.0011 (19)
O22	0.023 (2)	0.032 (2)	0.030 (2)	0.0016 (18)	0.0043 (18)	0.0011 (19)
S13	0.0094 (17)	0.0220 (16)	0.0132 (15)	0.0023 (12)	0.0022 (12)	−0.0002 (12)
O23	0.011 (2)	0.021 (2)	0.0093 (19)	0.0021 (17)	0.0047 (16)	0.0007 (16)
O24	0.011 (2)	0.021 (2)	0.0093 (19)	0.0021 (17)	0.0047 (16)	0.0007 (16)
O25	0.011 (2)	0.021 (2)	0.0093 (19)	0.0021 (17)	0.0047 (16)	0.0007 (16)
O26	0.011 (2)	0.021 (2)	0.0093 (19)	0.0021 (17)	0.0047 (16)	0.0007 (16)
O25'	0.011 (2)	0.021 (2)	0.0093 (19)	0.0021 (17)	0.0047 (16)	0.0007 (16)
O26'	0.011 (2)	0.021 (2)	0.0093 (19)	0.0021 (17)	0.0047 (16)	0.0007 (16)
S14	0.0185 (19)	0.0208 (16)	0.0233 (18)	0.0006 (14)	0.0023 (14)	−0.0053 (13)
O27	0.026 (3)	0.042 (3)	0.044 (3)	−0.003 (2)	0.003 (2)	−0.007 (2)
O28	0.026 (3)	0.042 (3)	0.044 (3)	−0.003 (2)	0.003 (2)	−0.007 (2)
O29	0.026 (3)	0.042 (3)	0.044 (3)	−0.003 (2)	0.003 (2)	−0.007 (2)
O30	0.026 (3)	0.042 (3)	0.044 (3)	−0.003 (2)	0.003 (2)	−0.007 (2)

### Geometric parameters (Å, º)

**Table:**

Cd1—O2	2.323 (7)	C7—N14	1.334 (13)
Cd1—O3	2.350 (7)	N9—H9A	0.8800
Cd1—O4	2.367 (10)	N9—H9B	0.8800
Cd1—O1	2.423 (9)	N10—H10A	0.8800
Cd1—S1	2.580 (4)	N10—H10B	0.8800
Cd1—S2	2.598 (4)	N11—H11A	0.8800
S1—C1	1.743 (16)	N11—H11B	0.8800
S2—C2	1.718 (9)	N12—H12A	0.8800
C1—N2	1.312 (18)	N12—H12B	0.8800
C1—N1	1.32 (2)	N13—H13A	0.8800
C2—N4	1.314 (13)	N13—H13B	0.8800
C2—N3	1.325 (12)	N14—H14A	0.8800
N1—H1A	0.8800	N14—H14B	0.8800
N1—H1B	0.8800	O9—H91	0.8206
N2—H2A	0.8800	O9—H92	0.8215
N2—H2B	0.8800	O10—H101	0.8138
N3—H3A	0.8800	O10—H102	0.8211
N3—H3B	0.8800	O11—H111	0.8227
N4—H4A	0.8800	O11—H112	0.8191
N4—H4B	0.8800	Cd4—O12	2.303 (7)
O1—H11	0.8191	Cd4—O14	2.422 (9)
O1—H12	0.8237	Cd4—O13	2.452 (9)
O2—H21	0.8211	Cd4—S10	2.566 (4)
O2—H22	0.8219	Cd4—S9	2.597 (3)
O3—H31	0.8252	Cd4—S8	2.705 (3)
O3—H32	0.8223	S8—C8	1.757 (10)
O4—H41	0.8216	S9—C9	1.752 (10)
O4—H42	0.8133	S10—C10	1.733 (15)
Cd2—O8	2.355 (11)	C8—N15	1.293 (14)
Cd2—O6	2.362 (8)	C8—N16	1.319 (14)
Cd2—O7	2.364 (8)	C9—N18	1.301 (13)
Cd2—O5	2.398 (10)	C9—N17	1.311 (13)
Cd2—S4	2.580 (4)	C10—N20	1.322 (18)
Cd2—S3	2.585 (4)	C10—N19	1.32 (2)
S3—C3	1.731 (11)	N15—H15A	0.8800
S4—C4	1.726 (15)	N15—H15B	0.8800
C3—N5	1.314 (13)	N16—H16A	0.8800
C3—N6	1.320 (13)	N16—H16B	0.8800
C4—N8	1.312 (19)	N17—H17A	0.8800
C4—N7	1.313 (18)	N17—H17B	0.8800
N5—H5A	0.8800	N18—H18A	0.8800
N5—H5B	0.8800	N18—H18B	0.8800
N6—H6A	0.8800	N19—H19A	0.8800
N6—H6B	0.8800	N19—H19B	0.8800
N7—H7A	0.8800	N20—H20A	0.8800
N7—H7B	0.8800	N20—H20B	0.8800
N8—H8A	0.8800	O12—H121	0.8195
N8—H8B	0.8800	O12—H122	0.8146
O5—H51	0.8497	O13—H131	0.8174
O5—H52	0.8208	O13—H132	0.8223
O6—H61	0.8232	O14—H141	0.8179
O6—H62	0.8253	O14—H142	0.8213
O7—H71	0.8188	S11—O16	1.461 (8)
O7—H72	0.8210	S11—O18	1.472 (8)
O8—H81	0.8250	S11—O17	1.484 (9)
O8—H82	0.8146	S11—O15	1.519 (8)
Cd3—O9	2.340 (8)	S12—O19	1.441 (10)
Cd3—O10	2.455 (9)	S12—O21	1.466 (11)
Cd3—O11	2.480 (10)	S12—O22	1.474 (9)
Cd3—S5	2.559 (4)	S12—O20	1.497 (9)
Cd3—S6	2.579 (3)	S13—O26'	1.427 (17)
Cd3—S7	2.700 (3)	S13—O25	1.444 (10)
S5—C5	1.719 (15)	S13—O24	1.448 (9)
S6—C6	1.724 (10)	S13—O23	1.468 (8)
S7—C7	1.709 (10)	S13—O26	1.532 (11)
C5—N10	1.32 (2)	S13—O25'	1.645 (18)
C5—N9	1.32 (2)	S14—O29	1.459 (10)
C6—N12	1.304 (13)	S14—O27	1.470 (11)
C6—N11	1.321 (13)	S14—O28	1.476 (10)
C7—N13	1.321 (14)	S14—O30	1.494 (9)
			
O2—Cd1—O3	77.0 (3)	N11—C6—S6	122.1 (8)
O2—Cd1—O4	94.3 (3)	N13—C7—N14	118.3 (9)
O3—Cd1—O4	78.3 (3)	N13—C7—S7	122.2 (8)
O2—Cd1—O1	81.0 (3)	N14—C7—S7	119.5 (8)
O3—Cd1—O1	153.0 (3)	C5—N9—H9A	120.0
O4—Cd1—O1	88.0 (3)	C5—N9—H9B	120.0
O2—Cd1—S1	166.8 (2)	H9A—N9—H9B	120.0
O3—Cd1—S1	114.2 (2)	C5—N10—H10A	120.0
O4—Cd1—S1	82.0 (3)	C5—N10—H10B	120.0
O1—Cd1—S1	86.2 (2)	H10A—N10—H10B	120.0
O2—Cd1—S2	99.9 (2)	C6—N11—H11A	120.0
O3—Cd1—S2	86.8 (2)	C6—N11—H11B	120.0
O4—Cd1—S2	156.5 (2)	H11A—N11—H11B	120.0
O1—Cd1—S2	112.5 (2)	C6—N12—H12A	120.0
S1—Cd1—S2	87.92 (13)	C6—N12—H12B	120.0
C1—S1—Cd1	104.8 (4)	H12A—N12—H12B	120.0
C2—S2—Cd1	109.9 (4)	C7—N13—H13A	120.0
N2—C1—N1	119.5 (14)	C7—N13—H13B	120.0
N2—C1—S1	122.0 (11)	H13A—N13—H13B	120.0
N1—C1—S1	118.6 (11)	C7—N14—H14A	120.0
N4—C2—N3	119.6 (9)	C7—N14—H14B	120.0
N4—C2—S2	121.2 (8)	H14A—N14—H14B	120.0
N3—C2—S2	119.2 (8)	Cd3—O9—H91	110.3
C1—N1—H1A	120.0	Cd3—O9—H92	116.7
C1—N1—H1B	120.0	H91—O9—H92	106.9
H1A—N1—H1B	120.0	Cd3—O10—H102	112.4
C1—N2—H2A	120.0	H101—O10—H102	107.7
C1—N2—H2B	120.0	Cd3—O11—H111	123.3
H2A—N2—H2B	120.0	Cd3—O11—H112	124.5
C2—N3—H3A	120.0	H111—O11—H112	106.9
C2—N3—H3B	120.0	O12—Cd4—O14	81.1 (3)
H3A—N3—H3B	120.0	O12—Cd4—O13	88.1 (3)
C2—N4—H4A	120.0	O14—Cd4—O13	91.4 (3)
C2—N4—H4B	120.0	O12—Cd4—S10	160.2 (2)
H4A—N4—H4B	120.0	O14—Cd4—S10	79.3 (2)
Cd1—O1—H11	112.7	O13—Cd4—S10	89.3 (2)
Cd1—O1—H12	127.8	O12—Cd4—S9	97.88 (19)
H11—O1—H12	106.9	O14—Cd4—S9	174.9 (2)
Cd1—O2—H21	99.3	O13—Cd4—S9	83.6 (2)
H21—O2—H22	106.9	S10—Cd4—S9	101.34 (13)
Cd1—O3—H31	104.9	O12—Cd4—S8	88.72 (19)
Cd1—O3—H32	137.0	O14—Cd4—S8	86.5 (2)
H31—O3—H32	106.4	O13—Cd4—S8	176.4 (2)
Cd1—O4—H41	118.2	S10—Cd4—S8	93.18 (12)
Cd1—O4—H42	97.6	S9—Cd4—S8	98.55 (11)
H41—O4—H42	107.7	C8—S8—Cd4	113.7 (3)
O8—Cd2—O6	95.5 (3)	C9—S9—Cd4	107.7 (4)
O8—Cd2—O7	78.0 (3)	C10—S10—Cd4	105.5 (5)
O6—Cd2—O7	75.3 (3)	N15—C8—N16	121.0 (9)
O8—Cd2—O5	90.4 (3)	N15—C8—S8	119.7 (8)
O6—Cd2—O5	79.3 (3)	N16—C8—S8	119.1 (8)
O7—Cd2—O5	150.8 (3)	N18—C9—N17	121.7 (9)
O8—Cd2—S4	82.8 (3)	N18—C9—S9	121.2 (8)
O6—Cd2—S4	165.6 (2)	N17—C9—S9	117.1 (8)
O7—Cd2—S4	118.0 (2)	N20—C10—N19	119.2 (14)
O5—Cd2—S4	86.3 (2)	N20—C10—S10	122.7 (12)
O8—Cd2—S3	154.4 (2)	N19—C10—S10	118.2 (11)
O6—Cd2—S3	99.1 (2)	C8—N15—H15A	120.0
O7—Cd2—S3	85.6 (2)	C8—N15—H15B	120.0
O5—Cd2—S3	112.8 (2)	H15A—N15—H15B	120.0
S4—Cd2—S3	87.91 (13)	C8—N16—H16A	120.0
C3—S3—Cd2	111.0 (4)	C8—N16—H16B	120.0
C4—S4—Cd2	105.0 (4)	H16A—N16—H16B	120.0
N5—C3—N6	118.7 (10)	C9—N17—H17A	120.0
N5—C3—S3	119.8 (8)	C9—N17—H17B	120.0
N6—C3—S3	121.5 (8)	H17A—N17—H17B	120.0
N8—C4—N7	120.6 (13)	C9—N18—H18A	120.0
N8—C4—S4	118.3 (11)	C9—N18—H18B	120.0
N7—C4—S4	121.1 (11)	H18A—N18—H18B	120.0
C3—N5—H5A	120.0	C10—N19—H19A	120.0
C3—N5—H5B	120.0	C10—N19—H19B	120.0
H5A—N5—H5B	120.0	H19A—N19—H19B	120.0
C3—N6—H6A	120.0	C10—N20—H20A	120.0
C3—N6—H6B	120.0	C10—N20—H20B	120.0
H6A—N6—H6B	120.0	H20A—N20—H20B	120.0
C4—N7—H7A	120.0	Cd4—O12—H121	121.2
C4—N7—H7B	120.0	H121—O12—H122	107.9
H7A—N7—H7B	120.0	Cd4—O13—H131	115.0
C4—N8—H8A	120.0	Cd4—O13—H132	126.2
C4—N8—H8B	120.0	H131—O13—H132	107.3
H8A—N8—H8B	120.0	Cd4—O14—H141	119.0
Cd2—O5—H51	123.5	Cd4—O14—H142	102.3
Cd2—O5—H52	129.5	H141—O14—H142	107.3
H51—O5—H52	104.9	O16—S11—O18	110.8 (5)
Cd2—O6—H61	103.5	O16—S11—O17	109.8 (5)
Cd2—O6—H62	127.4	O18—S11—O17	109.4 (5)
H61—O6—H62	106.4	O16—S11—O15	111.9 (4)
Cd2—O7—H72	110.7	O18—S11—O15	107.1 (4)
H71—O7—H72	107.5	O17—S11—O15	107.8 (5)
Cd2—O8—H81	134.9	O19—S12—O21	110.6 (6)
H81—O8—H82	107.1	O19—S12—O22	111.6 (5)
O9—Cd3—O10	87.9 (3)	O21—S12—O22	110.9 (6)
O9—Cd3—O11	79.7 (3)	O19—S12—O20	106.7 (5)
O10—Cd3—O11	85.6 (3)	O21—S12—O20	109.3 (6)
O9—Cd3—S5	158.8 (2)	O22—S12—O20	107.5 (5)
O10—Cd3—S5	87.8 (2)	O26'—S13—O25	75.7 (8)
O11—Cd3—S5	79.3 (3)	O26'—S13—O24	114.5 (8)
O9—Cd3—S6	97.1 (2)	O25—S13—O24	116.0 (6)
O10—Cd3—S6	85.1 (2)	O26'—S13—O23	122.2 (8)
O11—Cd3—S6	170.2 (2)	O25—S13—O23	112.9 (5)
S5—Cd3—S6	103.29 (13)	O24—S13—O23	111.3 (5)
O9—Cd3—S7	88.8 (2)	O25—S13—O26	107.6 (6)
O10—Cd3—S7	173.9 (2)	O24—S13—O26	105.2 (5)
O11—Cd3—S7	88.7 (2)	O23—S13—O26	102.6 (5)
S5—Cd3—S7	93.29 (12)	O26'—S13—O25'	109.9 (9)
S6—Cd3—S7	100.53 (12)	O24—S13—O25'	99.6 (7)
C5—S5—Cd3	105.9 (5)	O23—S13—O25'	94.9 (7)
C6—S6—Cd3	107.3 (4)	O26—S13—O25'	141.6 (8)
C7—S7—Cd3	113.8 (4)	O29—S14—O27	111.1 (6)
N10—C5—N9	119.0 (14)	O29—S14—O28	109.9 (6)
N10—C5—S5	122.0 (11)	O27—S14—O28	108.7 (6)
N9—C5—S5	118.8 (11)	O29—S14—O30	108.8 (5)
N12—C6—N11	119.1 (10)	O27—S14—O30	108.0 (6)
N12—C6—S6	118.7 (8)	O28—S14—O30	110.2 (6)
			
O2—Cd1—S1—C1	71.6 (11)	O9—Cd3—S6—C6	−42.3 (4)
O3—Cd1—S1—C1	−140.9 (5)	O10—Cd3—S6—C6	−129.6 (4)
O4—Cd1—S1—C1	145.8 (6)	S5—Cd3—S6—C6	143.8 (4)
O1—Cd1—S1—C1	57.3 (6)	S7—Cd3—S6—C6	47.9 (4)
S2—Cd1—S1—C1	−55.4 (5)	O9—Cd3—S7—C7	42.6 (4)
O2—Cd1—S2—C2	20.4 (4)	O11—Cd3—S7—C7	122.3 (5)
O3—Cd1—S2—C2	−55.8 (4)	S5—Cd3—S7—C7	−158.6 (4)
O4—Cd1—S2—C2	−106.0 (8)	S6—Cd3—S7—C7	−54.4 (4)
O1—Cd1—S2—C2	104.7 (5)	Cd3—S5—C5—N10	35.1 (12)
S1—Cd1—S2—C2	−170.2 (4)	Cd3—S5—C5—N9	−148.8 (10)
Cd1—S1—C1—N2	−36.1 (12)	Cd3—S6—C6—N12	−149.3 (8)
Cd1—S1—C1—N1	144.1 (10)	Cd3—S6—C6—N11	34.3 (10)
Cd1—S2—C2—N4	26.5 (10)	Cd3—S7—C7—N13	−80.8 (9)
Cd1—S2—C2—N3	−156.1 (7)	Cd3—S7—C7—N14	102.4 (9)
O8—Cd2—S3—C3	102.8 (8)	O12—Cd4—S8—C8	−42.8 (5)
O6—Cd2—S3—C3	−21.4 (4)	O14—Cd4—S8—C8	−124.0 (5)
O7—Cd2—S3—C3	53.0 (4)	S10—Cd4—S8—C8	156.9 (4)
O5—Cd2—S3—C3	−103.6 (5)	S9—Cd4—S8—C8	55.0 (4)
S4—Cd2—S3—C3	171.3 (4)	O12—Cd4—S9—C9	39.0 (4)
O8—Cd2—S4—C4	−150.5 (6)	O13—Cd4—S9—C9	126.1 (4)
O6—Cd2—S4—C4	−66.4 (11)	S10—Cd4—S9—C9	−145.9 (4)
O7—Cd2—S4—C4	137.2 (5)	S8—Cd4—S9—C9	−50.9 (4)
O5—Cd2—S4—C4	−59.7 (5)	O12—Cd4—S10—C10	138.2 (8)
S3—Cd2—S4—C4	53.3 (5)	O14—Cd4—S10—C10	147.4 (6)
Cd2—S3—C3—N5	157.4 (8)	O13—Cd4—S10—C10	55.9 (6)
Cd2—S3—C3—N6	−21.8 (10)	S9—Cd4—S10—C10	−27.4 (6)
Cd2—S4—C4—N8	−147.2 (10)	S8—Cd4—S10—C10	−126.8 (6)
Cd2—S4—C4—N7	35.7 (11)	Cd4—S8—C8—N15	−103.8 (8)
O9—Cd3—S5—C5	−136.5 (7)	Cd4—S8—C8—N16	80.9 (8)
O10—Cd3—S5—C5	−57.8 (6)	Cd4—S9—C9—N18	−32.9 (9)
O11—Cd3—S5—C5	−143.8 (6)	Cd4—S9—C9—N17	147.3 (7)
S6—Cd3—S5—C5	26.6 (5)	Cd4—S10—C10—N20	−32.0 (13)
S7—Cd3—S5—C5	128.2 (5)	Cd4—S10—C10—N19	147.2 (10)

### Hydrogen-bond geometry (Å, º)

**Table:**

*D*—H···*A*	*D*—H	H···*A*	*D*···*A*	*D*—H···*A*
N1—H1*A*···O26^i^	0.88	2.14	2.958 (16)	154
N1—H1*A*···O26′^i^	0.88	2.24	3.11 (2)	168
N1—H1*B*···O17^i^	0.88	2.10	2.927 (15)	156
N1—H1*B*···O15^i^	0.88	2.55	3.111 (14)	122
N2—H2*A*···O24^i^	0.88	2.08	2.934 (15)	163
N2—H2*B*···O1	0.88	2.02	2.875 (14)	165
N3—H3*A*···O27	0.88	2.14	3.014 (14)	169
N4—H4*A*···O28	0.88	2.17	3.000 (13)	157
N4—H4*B*···O3	0.88	2.21	3.028 (12)	155
N4—H4*B*···O2	0.88	2.63	3.183 (12)	121
O1—H11···O29^ii^	0.82	1.96	2.743 (13)	160
O1—H12···O14	0.82	1.97	2.770 (12)	164
O2—H21···O26^ii^	0.82	2.05	2.636 (12)	128
O2—H21···O26′^ii^	0.82	2.12	2.856 (18)	150
O3—H32···O18^ii^	0.82	1.91	2.679 (10)	156
O4—H41···O15^ii^	0.82	1.88	2.691 (13)	172
O4—H42···O29^ii^	0.81	2.16	2.973 (12)	173
N5—H5*A*···O24^i^	0.88	2.13	3.000 (11)	171
N6—H6*A*···O23^i^	0.88	2.14	2.986 (11)	162
N6—H6*B*···O7	0.88	2.17	2.982 (12)	153
N6—H6*B*···O6	0.88	2.59	3.157 (12)	123
N7—H7*A*···O27	0.88	2.08	2.938 (17)	165
N7—H7*B*···O5	0.88	2.06	2.915 (14)	163
N8—H8*A*···O30	0.88	2.14	2.989 (15)	162
N8—H8*B*···O21^iii^	0.88	2.10	2.932 (15)	159
N8—H8*B*···O19^iii^	0.88	2.65	3.223 (14)	124
O5—H52···O11	0.82	2.09	2.887 (13)	164
O5—H51···N7	0.85	2.46	2.915 (14)	114
O6—H62···O30^iv^	0.83	1.84	2.641 (11)	162
O7—H72···O22^v^	0.82	1.95	2.718 (11)	155
O8—H81···O16^iv^	0.83	1.96	2.756 (12)	162
O8—H82···O10^iii^	0.81	2.18	2.922 (13)	152
N9—H9*A*···O21	0.88	2.40	3.150 (17)	143
N9—H9*A*···O20	0.88	2.50	3.320 (16)	156
N9—H9*B*···O27	0.88	1.98	2.834 (17)	164
N10—H10*A*···O21	0.88	2.26	3.040 (17)	148
N10—H10*B*···O10	0.88	2.10	2.954 (15)	163
N11—H11*A*···O18^i^	0.88	1.98	2.855 (12)	177
N11—H11*B*···O9	0.88	2.13	2.994 (12)	167
N12—H12*A*···O17^i^	0.88	1.93	2.797 (12)	169
N13—H13*A*···O2^iv^	0.88	2.36	3.193 (11)	158
N13—H13*B*···O28^iv^	0.88	1.98	2.848 (13)	168
N14—H14*A*···O26^i^	0.88	2.22	2.980 (13)	145
O9—H91···O28^iv^	0.82	2.03	2.810 (12)	159
O9—H92···O20^iv^	0.82	1.94	2.721 (11)	159
O9—H92···O22^iv^	0.82	2.54	3.189 (10)	137
O10—H101···O9	0.81	2.67	3.331 (11)	139
O10—H102···O25′^iv^	0.82	1.77	2.57 (2)	164
O10—H102···O25^iv^	0.82	1.99	2.761 (13)	156
O11—H111···O25^iv^	0.82	2.15	2.851 (14)	143
O11—H111···O26′^iv^	0.82	2.18	2.972 (18)	163
O11—H112···O29^iv^	0.82	1.96	2.718 (13)	153
N15—H15*A*···O30	0.88	2.44	3.222 (12)	148
N15—H15*A*···N8	0.88	2.69	3.331 (15)	131
N16—H16*A*···O6^vi^	0.88	2.34	3.184 (11)	161
N16—H16*B*···O23^ii^	0.88	1.99	2.866 (11)	172
N17—H17*A*···O21^iii^	0.88	1.99	2.864 (13)	176
N18—H18*A*···O22^iii^	0.88	1.98	2.846 (12)	169
N18—H18*B*···O12	0.88	2.15	2.998 (11)	163
N19—H19*A*···O17^vii^	0.88	2.42	3.174 (15)	145
N19—H19*A*···O16^vii^	0.88	2.43	3.258 (15)	156
N19—H19*B*···O24^i^	0.88	2.03	2.887 (15)	163
N19—H19*B*···O25′^i^	0.88	2.65	3.27 (2)	129
N20—H20*A*···O17^vii^	0.88	2.21	3.018 (16)	153
N20—H20*B*···O13	0.88	2.11	2.966 (15)	165
O12—H121···O23^ii^	0.82	2.04	2.846 (10)	167
O12—H122···O16^viii^	0.81	2.06	2.740 (9)	140
O13—H131···O19^ii^	0.82	1.88	2.694 (12)	171
O13—H132···O4^iii^	0.82	2.23	2.981 (12)	153
O14—H141···O20^ii^	0.82	1.90	2.714 (11)	173
O14—H142···O25^ii^	0.82	2.10	2.750 (14)	135
O14—H142···O25′^ii^	0.82	2.00	2.79 (2)	161

Symmetry codes: (i) *x*+1, −*y*+1, *z*−1/2; (ii) *x*, −*y*+1, *z*−1/2; (iii) *x*, *y*+1, *z*; (iv) *x*+1, *y*, *z*; (v) *x*+1, *y*+1, *z*; (vi) *x*−1, *y*, *z*; (vii) *x*+1, −*y*+2, *z*−1/2; (viii) *x*, −*y*+2, *z*−1/2.
